# Virtual Care Perceptions and Experiences of Older Adults During COVID-19 in Canada: A Systematic Review

**DOI:** 10.3390/healthcare13151937

**Published:** 2025-08-07

**Authors:** Donna Gao, Angela Xu, Lixia Yang

**Affiliations:** 1 Michael G. DeGroote School of Medicine, McMaster University, Hamilton, ON L8S 4L8, Canada; 2 Department of Epidemiology and Biostatistics, Western University, London, ON N6G 3K7, Canada; 3 Department of Psychology, Toronto Metropolitan University, Toronto, ON M5B 2K3, Canada

**Keywords:** older adults, Canada, virtual care, barriers, COVID-19, socioeconomic disparities, technology, support

## Abstract

**Background/Objectives**: Older adults (65+) are the fastest growing age group in Canada, comprising 18.8% of the country’s population. During the COVID-19 pandemic, use of virtual care, including telehealth and tele-medicine, increased dramatically among older adults in Canada who often face higher health risks, mobility limitations, and many barriers to accessing healthcare. Despite the rapid expansion in virtual care, no systematic review has focused specifically on virtual care among older adults in Canada. This review aims to explore the factors influencing virtual care adoption and the experiences of older Canadians during the pandemic through a systematic review. **Methods**: Conducted in accordance with the Preferred Reporting Items for Systematic reviews and Meta-Analyses (PRISMA) guidelines, the review involved a comprehensive search of PubMed, Scopus, ESCBOHost, and Web of Science on 2 May 2025, yielding 281 unique citations. After screening and applying eligibility criteria, 15 studies employing quantitative, qualitative, or mixed-methods designs, with sample sizes ranging from 15 to 2,282,798, were included and appraised using the Mixed Methods Appraisal Tool (MMAT). **Results**: The review identified three domains of factors and the ways in which each factor shapes older adults’ virtual care experiences: (1) personal factors influencing virtual care use and demand (e.g., age, education, language, income, immigration status, community sizes), (2) resource factors impacting virtual care adoption (e.g., technology access, support), and (3) varying virtual care experiences among older adults (e.g., in assessment and communication efficacy, privacy, care quality, convenience, safety, and costs). **Conclusions**: This review highlights the complexities of virtual care engagement among older adults and underscores the need for inclusive, tailored strategies to improve the accessibility and effectiveness of virtual care delivery in both pandemic and post-pandemic contexts.

## 1. Introduction

Older adults over the age of 65 are the fastest growing age group in Canada. During the COVID-19 pandemic, virtual care use increased dramatically among older adults in Canada who often face higher health risks, mobility limitations, and barriers to accessing healthcare. Virtual care, including telemedicine and telehealth, is defined in this paper as “interactions between patients and/or members of their circle of care, occurring remotely, using any forms of communication or information technologies, with the aim of facilitating and maximizing the quality and effectiveness of patient care” [1]. These interactions can be conducted via secure messaging, email, telephone consultations, and secure video conferencing [1,2]. In response to the COVID-19 pandemic, virtual care has seen a dramatic increase in adoption due to its ability to provide medical and health services efficiently while maintaining social distancing measures [3]. Virtual care use and interest has surged both in Canada and globally. In the early phase of the pandemic, virtual care use increased 56-fold, accounting for about 71% of primary care physician visits in Ontario, Canada [4]. Virtual visits by cancer patients in the Princess Margaret Cancer Centre, Ontario, increased from 0.8% to 68.4% since the start of the pandemic [5]. Similar trends of virtual care use have been seen in past health-crises globally, including the Severe Acute Respiratory Syndrome (SARS) pandemic in 2003 in China [6], and various natural disasters that led to patient displacement [3].

In Canada, virtual care use is extremely important to the older adult population, defined in this paper as adults 65 years or older. They comprise over 18.8%, a significant portion, of the total Canadian population that is rapidly growing [7]. Older adults in Ontario had some of the highest proportions of virtual care use compared to other age groups, with virtual care constituting 73.4% of overall primary care visits in the 65–74 year age group, and 72.5% for adults aged 75+ in the early pandemic period [8]. Primary care plays a pivotal role in the Canadian healthcare system and many other health systems worldwide [8,9]. Older adults rely heavily on these primary health services due to higher prevalence of health issues and concerns [10,11]. Regarding virtual care, the older adult population has unique characteristics and demands that may change their experiences relative to other age groups. For example, the majority of older adults suffer from chronic health conditions, which require longitudinal management [12]. Additionally, barriers to technology use, such as digital literacy and access to digital devices, may be more pronounced in older adult populations [9,13]. In 2018,According to the latest data prior to the pandemic, in 2018, only 71.2% of the older adult population in Canada had used the internet in the past 3 months, compared to 91.3% for the general population above 15 years of age [14]. Canada’s older adult population also consists of a large proportion (around 30%) of immigrants [4]. A scoping review of older immigrants’ access to primary healthcare in Canada suggested multiple access barriers, including those related to language, culture, cost, insurance, availability, and location, that immigrants living and aging in a foreign country often experience [15]. Underutilization of medical and primary healthcare, health promotion and cancer screening and mental health services among older immigrants has been widely documented in Canada [2,13,15,16,17,18]. This indicates the importance of considering the spectrum of circumstances and challenges facing the highly heterogeneous older adult population when developing virtual care delivery strategies in Canada.

Although we are now in the post-pandemic era, virtual care use will likely continue, particularly as part of future emergency responses. For virtual care to be effective as part of an emergency response, it first needs to be routinely used in the healthcare system [3]. Proactive, rather than reactive implementation, is likely to generate benefits in the long-term, and help everyday and emergency healthcare challenges [3]. Literature suggests that patients should play a central role in developing policy recommendations for virtual care, but this reality has not yet been achieved [19,20,21]. This can be achieved by involving patients in research and using these findings to help inform policy making [20]. To date, there is no comprehensive systematic review with a specific focus on the experiences and outcomes in virtual care use among Canadian older adults. There exists a small number of reviews related to virtual care in broader regions and age groups. For example, a recent study reviewed 86 studies on telehealth for general healthcare or specific medical procedures to identify common challenges and motivations, but this review did not capture specific techniques or approaches to maximize virtual care use in older adults [22]. One systematic review [23] summarized 10 studies to determine telemedicine satisfaction among older adults, of which only one study pertained to Canadian patients. Similarly, another study [24] looked at the availability, application, and implementation of telemedicine in older adults, but did not include any Canadian studies. Although there is a growing output of virtual care literature in Canada [25], systematic reviews focusing on virtual care do not place an emphasis on older adults [26]. An understanding of the factors, experiences, and perceptions of virtual care utilization among Canada’s growing older adult population remains insufficiently explored and synthesized, highlighting a critical gap in understanding the nature and extent that virtual care, a critical phenomenon shaping today’s healthcare landscape, impacts older adult wellbeing and care delivery in Canada.

The purpose of this systematic review is to comprehensively identify and deepen the understanding of the perceptions and experiences of older adults in Canada with regard to the adoption of virtual care during the COVID-19 pandemic. By synthesizing the relevant findings from existing research and knowledge, this review aims to provide timely insights into the range of factors that shape the access and utilization of virtual care services among older adults in Canada. This information may be used to inform future policies on virtual care use and implementation, help address potential barriers and promote user-friendly virtual care strategies specific to this population in the future.

## 2. Materials and Methods

This review was conducted in accordance with Preferred Reporting Items for Systematic Reviews and Meta-Analysis (PRISMA) checklist [27]. The utilization of PRISMA facilitates a transparent, complete and accurate reporting of review results. Publications/articles were extracted from the following digital databases: PubMed, Scopus, ESCBOHost, and Web of Science. These databases capture a wide spectrum of medical and social science literature that allows for a comprehensive understanding of the topic.

### 2.1. Eligibility Criteria

The following inclusion criteria were used during the selection of articles for this review:

1. The article is in English, with full texts available electronically.
2. The article captures the experiences of older adults aged 65 and over in Canada.
3. The article focuses on virtual care during the COVID-19 pandemic, regardless of publication year.

Articles were excluded if they did not meet these inclusion criteria. Additionally, duplicates and articles that did not clearly identify their older adult participants were further excluded. Finally, commentaries, reviews, editorials, mathematical modeling reports, and methodological and conceptual papers were excluded as well.

### 2.2. Study Selection

A detailed search strategy was employed to cover articles encompassing terms in five identified domains: virtual care (and synonyms), barriers/challenges (and synonyms), COVID-19 (and synonyms), Canada, and older adults (and synonyms). For example, virtual care’s synonyms included several similar terms such as virtual medicine, telehealth, telemedicine, telecare, mobile health, mhealth, ehealth, and telerehabilitation. These five groups of keywords were searched with the Boolean operator AND between groups, and OR within synonyms in the same group. A detailed search strategy including search terms and search strings are included in Tables S1 and S2 in Supplementary File S1. A comprehensive literature search was conducted in the aforementioned databases, reflecting eligible literature published up to 2 May 2025, about two years since the WHO announced an end to COVID-19 as a public health emergency [28]. The timing of this systematic review allows for identification and inclusion of relevant studies due to the varying times (e.g., months to several years) it takes to publish scholarly articles that focus on the pandemic period (see eligibility criteria in Section 2.1).

### 2.3. Study Selection Process

The search and selection process included a systematic two-level approach: Level 1 (title and abstract) screening and Level 2 (full text) screening. All articles were managed using EndNote Web. Duplicate articles were removed prior to the scanning of article titles and abstracts, both manually and with the help of the EndNote Web software. Level 1 screening involved the collection of articles and scanning of the titles and abstracts to determine whether the concepts of Canadian older adult virtual care experiences during the COVID-19 pandemic were discussed to be within inclusion criteria. Subsequently, Level 2 screening involved a full text reading of the resulting articles to further determine their scope and relevance towards our topic. Level 2 screening was performed to identify the final articles included in the systematic review. Any articles that posed discrepancies towards inclusion or exclusion were screened using full text (Level 2).

The full text screening process involved having two reviews (the first and second author of this review) first independently assess all the sources before assessment results were compared and discussed. Any disagreement not resolved between the two reviews was then discussed with a third review (the third author). The screening process was able to successfully select articles that fit the aim of the systematic review and eligibility criteria.

### 2.4. Risk of Bias Assessment

The Mixed Methods Appraisal Tool (MMAT) was used to assess the included studies [29], a component in the PRISMA 2020 checklist. The MMAT allows critical appraisal of five categories of studies: qualitative research, randomized controlled trials, non-randomized studies, quantitative descriptive studies, and mixed methods studies [29]. The quality assessments of each article were conducted by the first two authors of this review, and any disagreements were discussed and resolved through consultation with the last author of the review.

## 3. Results

**Figure 1:**
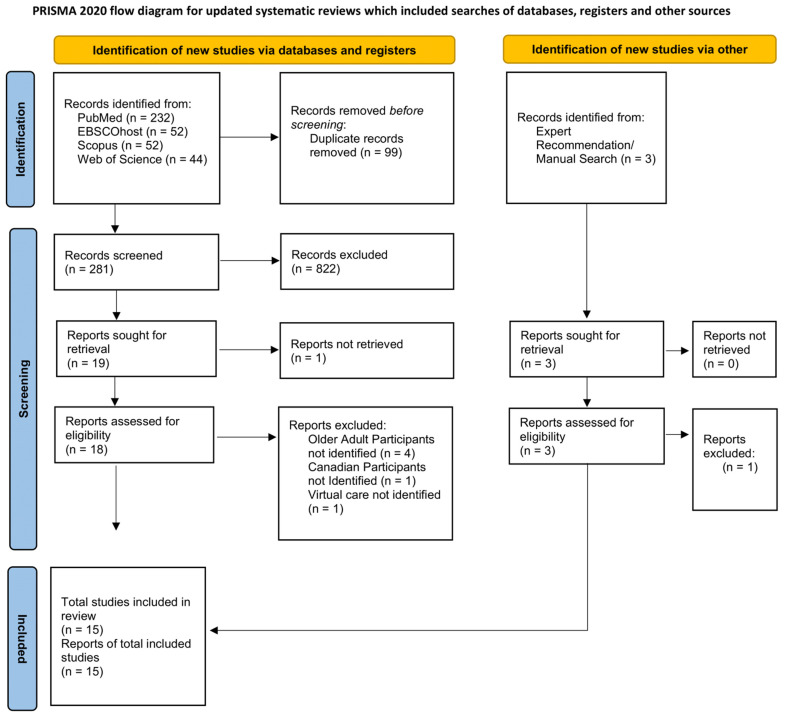
PRISMA 2020 flow diagram of the selection of studies regarding Virtual Care Perceptions and Experiences of Older Adults During COVID-19 Pandemic in Canada.

The PRISMA 2020 flow diagram (Figure 1) provides a visual representation of the selection process in extracting the final set of 15 articles included in this review. As suggested by the PRISMA 2020 checklist, the summary of main features of the selected individual studies is included in Table 1.

### 3.1. Studies Selected 

As illustrated in Figure 1, a total of 380 articles were extracted from the selected databases. After removing 99 duplicates, the titles and abstracts of the remaining 281 articles were further screened according to the inclusion criteria. This resulted in 19 articles, subjected to full text screening. Of the 19 articles, 1 article was not available in full text. The remaining 18 articles were screened, leaving 12 articles. Three additional articles were subsequently identified in a manual search. In total, a final sample of 15 articles were included in the analysis for this review.

### 3.2. Reporting Risk of Bias Assessment

Critical appraisal of each article’s methodological quality was conducted following the criteria specified in the MMAT guideline [29]. Each article is assessed based on the corresponding methodological quality criteria based on the category the article falls in—qualitative research, randomized controlled trials, non-randomized studies, quantitative descriptive studies, and mixed methods studies. As suggested by the MMAT tool, overall quality scores for the studies were not calculated and accumulated, but the quality of the studies was assessed against individual criterion [29]. The overall quality of the 15 articles included in this review is considered to be satisfactory, with identified limitations discussed later in the discussion section. The results of the quality appraisal are presented under Table S3 in Supplementary File S2.

### 3.3. Thematic Analysis of Results in Selected Studies

The selected studies in Table 1 revealed key factors relevant to older adults’ experiences with virtual care use in Canada. This systematic review first looked at barriers and facilitators to virtual care adoption, and uncovered factors in two main domains: (a). Personal factors impacting virtual care use and demand; (b). Resource factors impacting virtual care adoption. More specifically, personal factors include individual-specific or personal characteristics, including socioeconomic factors, that impact virtual care use. In particular, these personal factors help describe the demographic profiles of older adults who demand and use virtual care and are not as easily modifiable as resource factors. Resource factors refer to external environmental/situational factors such as technological infrastructure, financial resources, and environmental/situational factors such as technological infrastructure, and availability of external support. These elements play a crucial role in shaping the accessibility and effectiveness of virtual care services, and the presence or lack of these factors contribute to the facilitators or barriers of virtual care adoption. Finally, to understand the experiences of older adults who did use virtual care, we looked at outcomes and perceptions of virtual care experiences. These factors compile qualitative experiences of Canadian older adults using virtual care during the pandemic, offering insights into their healthcare interactions.

#### 3.3.1. Personal Factors Shaping Virtual Care Use and Demand

Four of the selected studies identified important socioeconomic factors of virtual care use and demand among older adults [5,11,31,41]. Among the four studies, one indicates age difference among older adults to be an important marker in virtual care use [11]. Virtual care visits were higher (90 visits per 1000) in older adults aged 75+, when compared to older adults aged 64–74 (75 visits per 1000), in Ontario [11]. Income impacted virtual care use. However, the relationship between income or socioeconomic status and virtual care usage is not always straightforward and consistent. For example, in one study, neighborhood income level was not revealed to be an influencing factor of telemedicine visits (e.g., telemedicine visits increasing at similar rates across all investigated income quintiles) [11]. In another two studies, however, lower household income (i.e., under $80,000 CAD per year) was found to be significantly associated with a lower use of video visits and a lower demand for all modes of virtual care (secure messaging, telephone, video) [41]; and 43% of the cancer patients who used virtual follow-ups had high household annual income (>$100,000), compared to 37% for those of low income (<$100,000) (20% no answer) [5]. Additionally, individuals with no private insurance had lower odds of using secure messaging, and expressed higher demand for telephone visits, which may have a lower barrier to entry [41].

Regarding education, older adults without an undergraduate degree were less likely to express a demand for all modes of virtual care (secure messaging, telephone, video) compared with those with an undergraduate degree or higher [41]. A higher use of telephone visits in particular was associated with higher education level. In [5], 68% of the cancer patients who used virtual follow ups had university education and above with a high health literacy; and 98% of them had at least one virtual visit via phone. When looking at digital health literacy as a whole, older adults with a higher digital health literacy expressed a higher demand and use of all modalities of telemedicine [41]. This may be emphasized with specific modalities of virtual care, for example, low digital literacy in older adults was identified as a barrier particularly for video-conferencing, which may require a higher level understanding of digital technologies compared to telephone-based communications [40].

The older adult population in Canada is a highly diverse group in culture and socioeconomic status. Older adult immigrants were found to have experienced significant disparities in virtual care use during the COVID-19 pandemic depending on their language ability, racial background, and immigration status [31,41]. Non-English speaking older adult immigrants expressed more demand for video visits and secure messaging, yet had a lower use of video visits or telephone visits [41]. Individuals born outside of Canada were less likely to use video visits but more likely to use secure messaging as a telemedicine channel compared to those born in Canada [41]. Non-white individuals expressed less demand for video visits and secure messaging but reported more use of telephone visits compared to white individuals [41]. At the beginning of the pandemic, older adult immigrants had a lower rate of virtual care use compared to non-immigrant counterparts (77; 86; per 1000), but as the pandemic progressed, the levels of use between the two groups became similar (80; 79; per 1000) [31]. Even though the study did not test the statistical significance of the differences, a converging pattern in virtual care rates between the two groups was evident [31]. It was also found that immigration classes played a role in the level of virtual care use. There was a lower level of virtual care use reported by older adult immigrants from the family class, in comparison to those from economic, refugee and other immigration classes [31]. Language barriers were reported by older adult patients who did not have proficiency in English or French [38]. Particularly, older adult immigrants fluent in English had higher levels of virtual care use relative to those not fluent in English and those fluent only in French [31].

Personal characteristics related to community size and residential location based on rurality were found to be linked to varying patterns of virtual care use. Prior to the pandemic, telemedicine uses for both rural and urban Ontarians were low, with a slightly higher rate of telemedicine visits in rural areas compared to urban areas [11]. During the pandemic, both urban and rural areas experienced a large surge in telemedicine use, with a significantly higher rate among urban (84 visits per 1000) than rural patients (54 per 1000) [11]. In a survey-based Canada wide study that defined rural community as a community with less than 1000 people, older adults residing in these remote rural areas had higher odds of using secure messaging for care during the pandemic compared to those living in small, medium, or large population centers and urban centers [41].

#### 3.3.2. Resource Factors Impacting Virtual Care Adoption

A number of studies in the review further explored specific resource factors influencing the adoption and utilization of virtual care for older adults in Canada. These factors included barriers and facilitators falling into two overarching categories: technology-related factors and support-related factors. Broadly speaking, technology-related factors include those related to the utilization, accessibility, or functionality of technology, whereas support-related factors encompass those facilitating the successful implementation and utilization of virtual care. The support can manifest as technological support, caregiver support, or educational support.

##### Technology-Related Factors

Technology is an essential component of virtual care. Virtual care cannot occur without technologies that allow for remote communication and connection. Insufficient access to technology is a direct barrier to virtual care adoption among older adults in multiple selected studies [30,32,39,40,42]. The access is demonstrated in physical devices and complementary components such as the internet, and adequate mobile or data plans [40]. Access to technology can be hindered in a variety of ways. The high cost associated with required technologies, such as iPads and mobile plans, was identified as a possible contributing factor for the insufficient access to virtual care [32,39,42,43]. Furthermore, technical difficulties were observed to diminish the effectiveness of virtual care use [39]. Ref. [42] Reported that older adult patients, caregivers, and healthcare providers all identified poor internet connection as a barrier for videoconferences. Additionally, poor signal or connections during telephone calls, resulting in an inability for the patient and healthcare provider to hear each other, was also identified as a technical difficulty encountered by many of the participants [32,39,42,43]. Compatibility issues between different software platforms and specific devices also emerged as another issue, particularly due to the extensive variety of virtual care platforms in use such as Jabber, EMR, and Webex [43].

Among older adults who had access to technology, proficiency in how to use the internet makes a difference in their virtual care experience. While 99% of the older cancer patients who used virtual care in [5], those who felt neutral, somewhat confident, or not confident using the internet for health-related purposes had a lower odds of being satisfied with virtual follow-ups compared to those who felt confident or very confident. Compared to internet-based videoconferencing appointments, there is a preference for telephone appointments among many older adults [5,30,32,34,35,38,39,42,43] due to several factors, including patient preference, internet connectivity challenges, particularly for older adults residing in rural areas, and lack of educational support [34].

##### Support-Related Factors

Another important component that influenced virtual care adoption and effectiveness was the presence (or absence) of support in a variety of settings. This includes technological support and caregiver support.

As previously emphasized, technological difficulties greatly hindered the effectiveness of virtual care use, posing a strong barrier to successful implementation of virtual care for older adults. Consequently, technological support (such as personnel and services) emerged as an important facilitator, with participants indicating its promising potential to enhance virtual care utilization [39,42,43].

Older adults often do not use virtual care in isolation, and the presence of caregivers, such as spouses, children, and other personal support all could facilitate virtual care visits [40,42]. Caregivers were able to help overcome some of the aforementioned barriers [42,43], with one study reporting that caregivers felt comfortable with basic set-up and troubleshooting of a virtual care visit [42].

#### 3.3.3. Perspectives of Virtual Care Experiences

Qualitative insights from a number of these studies [30,33,35,36,37,39,40,43] capture a comprehensive picture of older adults’ lived experiences in virtual care, particularly with regard to the outcomes after virtual care use. In this review, virtual care experiences are broadly categorized into perceptions of assessment efficacy, communication efficacy, resource expenditure, safety, and relationships.

##### Assessment Efficacy

Assessment efficacy referred to the perceptions on the effectiveness of virtual care. In particular, there were concerns about the lack of physical and tactile exams during the telemedicine care [30,32,36,37,38,40,43]. The general worry was that doctors would be more prone to accidentally missing something if the patient was not physically in front of them [30]. There were additional concerns regarding accuracy and effectiveness of virtual modalities [40,43] especially regarding complex medical cases, perceived emergencies, or initial consultations [38].

“...Sometimes, when you have a problem and you’re seeing a doctor, you want him to look, with his own eyeballs to see the actual thing. You…to see your skin, in the real thing not…not done through a camera, and you want him to poke you, you know, or feel. There’s so much in an examination, that should be done tactile, as opposed to only visual. Only visual, you miss so much without the tactile attached to it.”

[30]

##### Communication Efficacy

Communication efficacy captured perceptions on the effectiveness of communication between older adults and healthcare providers during virtual care visits. Notably, usual forms of virtual care (e.g., phone calls and video calls) largely eliminate nonverbal communication, such as facial expressions and body language, which negatively impacts the effectiveness and efficacy of communication [36,38,40]. Additionally, it has been reported that older adults with cognitive or hearing difficulties may find accessing virtual care especially difficult due to challenges in understanding and communication [30]. Overall, such communication challenges manifested as a concern over the ability for patients and doctors to express themselves via virtual modalities.

“…you do miss some of the eye contact and the body language, and I make the point that when people communicate, they often talk about the words only being about 7%, the tone being 38% of the…body language being 55%, so email or a phone, you might get the tone but you don’t get the body language and that’s, and, sometimes that’s very important. I know, how many times that I noticed body language, that I would ask another question, and bingo, the real problem would come out, where it wouldn’t have come up if you hadn’t been able to observe the body language.”

[30]

##### Resource Benefits

Perceptions on resource expenditure and convenience were also captured in the studies reviewed. In general, decreased resource expenditure, including reduced time and costs related to travel, parking, and waiting at the clinic were considered as a benefit of virtual care use [32,37]. Additionally, some older adults found virtual care to be convenient, as it does not involve travel and commute, particularly by older adults living outside of the city area or those who cannot leave their home [35,38,42]. Those who had ease of communication with care providers in virtual care settings also regarded virtual care to be convenient [40].

“It saves me the cost, time, and inconvenience of driving to and from the hospital, the cost of parking, and the long walk from the parking lot to the doctor’s office.”

[37]

##### Safety

Virtual care resulted in both positive and negative safety evaluations/perceptions. Some older adults felt positive about the reduced health risk towards contracting coronavirus, as they did not have to put their physical health at risk in a waiting room, high risk environment or appointment with a physician during the pandemic [30,36,38]. This also resulted in patients feeling less anxious about having to enter the hospital or clinics during the pandemic [37]. Conversely, some older adults did express concerns about safety, particularly regarding phone fraud for virtual visits conducted over the telephone [33]. Concerns were also expressed by healthcare providers regarding legal and professional liability issues in web-based medical platforms [38].

“It was much better during the COVID [pandemic] than having to go and sit in a waiting room with a whole bunch of other people even though you and they were masked.”

[36]

“Because there is nothing like the in-person kind of thing and when you’re talking over the phone again, you still have some privacy issues and there are some things that you prefer to have said completely in private.”

[34]

##### Social Relationships

Virtual care also impacted the physician-patient relationship, care coordination, and social activity in older adults’ lives. Regarding the physician-patient relationship, some older adults indicated that virtual care reduced the frequency of in-person interactions between the physician and patient, which may decrease the care continuity and coordination of physician activities [30]. Some noted that they were more comfortable with an in-person appointment with a specialist if it was their first time or did not already have an established relationship with the specialist [30,36]. Older adult patients in [35] noted the lack of emotional intimacy and lack of face-to-face contact during group activities in virtual cardiac rehabilitation, and their healthcare providers shared similar concern regarding decreasing trust that could negatively impact patient-provider relationships. Similarly, for telerehabilitation patients, having pre-established in-person early interactions was crucial in creating a positive virtual group dynamic [39]. Different from findings in [30,35,38], suggested that frequency of follow-ups in a telemedicine setting has increased during the pandemic, and that telemedicine indeed contributed to maintaining and improving the physician-patient relationship.

Virtual care may profoundly impact social activity and social connections for older adults. In person healthcare visits were identified as vital social experiences for older adults, particularly those who lived alone and already experienced isolation in their daily lives [30]. Switching to a virtual format removes much of the social activity and personal interactions, creating concerns over social isolation and exacerbating their feeling of loneliness during the pandemic [30].

“...for some people the doctor’s visit is one of your social experiences. The more you live alone, like I live alone, these kinds of contacts are part of your…socialization, your contacts…like going to the library, going to the doctor, these are all things where people have contact with others. So, if you make these things more virtual, you cut back on people’s contacts with the outside world.”

[30]

## 4. Discussion, Strengths and Limitations

### 4.1. Discussion of Results

The personal and resource factors identified in this review capture some of the general social determinants of health in older adults such as age, income and economic factors, social support, and availability of caregiving [44,45,46]. These factors are reflected in a more specific theoretical framework describing the social and digital determinants to equitable virtual care access during the COVID-19 pandemic in a report by Health Canada [47]. The personal factors identified from the review conform to the social determinants of virtual care access in [47] including age, socioeconomic, linguistic, and cultural factors, which have implications for digital health literacy, access to a safe place to have virtual consults, and access to high-quality broadband and technologies. In general, studies based in Canada and other countries suggest that the association between age and virtual care use could be due to increased health vulnerability of older adults (e.g., 75+), decreased mobility leading to preference to care from home, and higher health needs, and requiring more frequent healthcare access [11,48,49]. On the other hand, virtual care use potentially decreased in older adults with lower income. In particular, lower income was linked to fewer video medical visits, and this likely stems from challenges in accessing technology [48]. For example, the Canadian Internet Use Survey (CIUS) administered by Statistics Canada revealed that households with lower income had less access to the internet [50]. Additionally, the cost implications of setting up video capabilities for virtual care appointments may present a barrier compared to other virtual care options. Notably however, this finding was inconsistent across all studies in the review, with one study indicating that overall neighborhood income levels did not impact virtual care [11]. Our review finds that education less than an undergraduate degree level was associated with lower demand for all modes of virtual care. This is likely due to decreased digital health literacy, which was a key determinant in both virtual care use and demand. The close relationship between digital literacy and education is supported by a Canadian study of orthopedic patients that found that digital health literacy was lower in participants who did not attend university, compared to those who did [51]. Finally, older adult immigrants, as well as older adults who lack proficiency in English had lower virtual care use. Ethnic and racial disparities have also been reported. A cross-sectional survey of older adults at the Kaiser Permanente Medical Care program found that Black, Latino and Filipino older adults were less likely than White and Chinese older adults to own mobile devices and have the skills and experience to take advantage of mobile based tools [52].

The resource factors identified in this review parallel the digital determinants of virtual care access in [47], including structural (e.g., governance, policies, regulations) and technical (e.g., interoperability, connectivity) factors that have implications for equity, and have the tendency to be additive, or amplify inequities born out of social determinants of health [47,53]. The literature suggests that technology-related barriers may be compounded by socioeconomic disparities [32,39,42,43]. For example, older adults in some rural areas, or of lower socioeconomic status, may not have the capability to access good internet connectivity or technology [47,53,54]. Although structural factors were not explicitly found in this review, technical factors were recognized in multiple reviews. Consistent with previous literature, connectivity challenges, reduced access to technology, and lack of support have been identified in this review as major access barriers to virtual care [47,55,56]. Support may take the form of caregivers who can help facilitate virtual care use, education resources to help build digital literacy, and technological support to help overcome technological malfunctions. Previous literature studying American older adults have shown that other factors, such as computer anxiety, self-efficacy, and cognitive abilities also influence technology uptake in older adults [48,57]. Given this, support systems are even more critical, and may help decrease resistance to virtual care adoption in these populations [37,48]. Another study [58] suggested that ageism in the design process of digital technology may play a role as a possible barrier to technology adoption, with negative stereotypes of older adults’ technological abilities resulting in their exclusion in technology research and design [58]. Improving the inclusion and engagement of older adults in digital technology by investigating and acknowledging their needs serves as an important step to successful virtual care implementation. In addition, some general considerations for older adults who have little or no experience using technology include: (a) Using technology the individual is already familiar with, (b). Providing training/directions for use, as well as other print materials with instructions, reminders, and tips for technology use and troubleshooting, and (c). Involvement of a live-in family member or caretaker to help assist with any issues [58].

In the review, the main concerns of older adults who used virtual care included decreased assessment efficacy due to the absence of physical exams, concerns for virtual safety, and decreased communication efficacy due to elimination of non-verbal communication. As physical examinations are an important aspect of a healthcare interaction that are not readily accommodated by virtual care, it is unsurprising that this view has been noted in other reviews of virtual care as well [38,43,59]. A 2021 national survey of Canadian physicians underscored the significance of this issue by revealing that 80% of clinicians encountered difficulties with virtually examining patients, thereby emphasizing the magnitude of this problem [60]. Notably, this issue is not as simply resolved by using videoconferencing, as there could be reluctance, or lack of privacy, to use videoconferencing to show certain parts of the body [61]. This leads into another issue identified in this review. One paper identified that older adults may be concerned about phone fraud, and other similar papers have identified similar concerns about privacy or confidentiality across multiple user groups [42,43]. Some factors that could contribute to this include a perceived intrusiveness or lack of transparency regarding virtual care [55], as well as the fact that some patients and providers may not have the capability to have a quiet, confidential conversation with a healthcare provider from their home [36,62,63]. In addition, while support from family or caregivers may be an important facilitator in virtual care set-up and troubleshooting, it is still important that older adult patients are provided the privacy needed during their personal conversations [55]. As with all forms of healthcare, informed consent is important, and providers should stay up to date with the most recent local and federal frameworks and regulations [55,64]. Next, the elimination of nonverbal communication, including facial expressions and body language, decreased the effectiveness of communication between patient and provider [35]. Notably, this sentiment is expressed across multiple different types of virtual care modalities (telephone, videoconferencing) [36]. This could impact the physician-patient relationship. A systematic review [62] found that clinicians reported a decrease in their ability to develop and maintain a strong therapeutic relationship with service users. They struggled to interpret nonverbal cues and felt disconnected from their patients [62].

Despite these concerns, it should be noted that most older adults did have a general positive experience with virtual care and could be interested in pursuing it further in the future [42,43]. Multiple studies conducted both before and during the pandemic have also reported evidence of the effectiveness of virtual care in reducing treatment gaps and improving access to healthcare [54,62]. Positive advantages identified in this review include reduced health risk due to decreased exposure to and increased convenience due to decreased resource expenditure. Although these perceptions were taken during the COVID-19 pandemic, given the vulnerability of older adults to other infections (such as the flu, pneumonia, and antibiotic resistance bacteria), many of these advantages, including convenience, ease of visits, efficiency, and comfort are likely to persist in this age group post pandemic [37]. A hybrid approach that combines in-person and virtual care can leverage the strengths of both modalities while mitigating their respective limitations [35,38].

### 4.2. Strengths and Limitations

Following the PRISMA 2020 Checklist, this review identifies the influencing personal and resource factors, as well as outcomes and perspectives of older adults in Canada with regard to the adoption of virtual care during the COVID-19 pandemic. By focusing on this specific demographic, the review fills a notable gap in the literature, offering a comprehensive understanding of their unique perspectives on virtual care and adding to the theoretical framework of the social determinants of health in older adults. Additionally, the perceptions and challenges of virtual care were captured not only from the perspectives of elderly patients but also from their caregivers and healthcare provider teams, offering views from multiple influencing parties within the context of older adults in Canada during the COVID-19 pandemic.

However, it is important to consider certain limitations when interpreting the findings of this review. The generalizability of the review may be limited due to the unequal representation of provinces and territories in Canada, with the majority of studies focusing on Ontario, and several on the Toronto areas. While Ontario does constitute a significant portion of Canada’s population, this focus may not fully capture perspectives from other provinces, territories and less urbanized regions. Additionally, the methodological constraints used in the searches excluded non-English and non-French speakers, potentially reducing the representativeness and generalizability of the findings, including those related to immigrant and ethnic minority groups. The search terms in this review were carefully selected and thoroughly discussed based on the research aim. The findings from the review indicates additional search terms could be included in some domains. For example, “facilitator” and “strategy” could be added in the “barriers/challenge” domain, as some studies discussed both barriers and facilitators at the same time. Finally, extracting and synthesizing qualitative data can be subjective and prone to bias. Subconscious biases and author perspectives may influence how the findings of this paper were selected, analyzed, and reported.

## 5. Conclusions

This systematic review has identified key factors influencing older adults’ experiences with virtual care in Canada amidst the COVID-19 pandemic. Personal factors highlight possible socioeconomic disparities in access. Resource factors, such as access to technology and the need for support, are identified as important facilitators or barriers to adoption. Finally, stakeholder experiences underscore ongoing concerns regarding assessment efficacy, communication, and patient privacy. Despite the challenges, however, virtual care offers many benefits, such as convenience, increased safety, and decreased resource expenditure for many users in a variety of care settings including primary care, telerehabilitation and cancer care. Continued efforts to optimize its effectiveness and accessibility for this important demographic, both during and beyond the COVID-19 pandemic, are vastly beneficial for improving the wellbeing of older adults as well as enhancing care delivery by the healthcare system. In this regard, by summarizing older adults’ experiences with virtual care, this paper helps inform policy making, ensuring that future virtual care systems are oriented around and responsive to patients’ perspectives [20]. The paper provides timely implications for developing evidence-based strategies to improve virtual healthcare access and utilization, maintain continuity of care, and advance equitable healthcare delivery. These strategies and initiatives can be implemented through effectively integrating virtual care into comprehensive care models, for example, a hybrid care system that combines face-to-face in-person care and virtual visits, that will better meet the diverse needs of the rapidly growing older adult populations in Canada.

## Figures and Tables

**Table 1:** Summary of the key features of the selected studies.

Study	Country/Study Area	Article Title	Methods	Population/Subgroup
[30]	Alberta, Canada Ontario, Canada	The Impact of COVID-19 on Older Adults’ Perceptions of Virtual Care: Qualitative Study	Qualitative: Semi structured interviews of participants (n = 20)	Older adults (65 years and above) Rural, urban, suburban, BIPOC, white
[5]	Toronto, Ontario, Canada	Virtual follow-up care among breast and prostate cancer patients during and beyond the COVID-19 pandemic: Association with distress	Quantitative: cross-sectional virtual care evaluation survey (n = 352)	Older adult breast and prostate cancer patients (average age 65 years) receiving virtual care.
[31]	Ontario, Canada	Virtual Care Use Among Older Immigrant Adults in Ontario, Canada during the COVID19 Pandemic: A Repeated Cross-Sectional Analysis	Population-based, repeated cross-sectional study (n = 2,282,798)	Virtual ambulatory visits among older immigrants (age 65 years and older) residing in Ontario, Canada with valid Ontario Health Insurance Plan (OHIP) coverage
[11]	Ontario, Canada	The Use of Telemedicine in Older-Adults During the COVID-19 Pandemic: a Weekly Cross-Sectional Analysis in Ontario, Canada	Quantitative: Cross-sectional study using administrative data (n = 2,282,798)	Older adults (65 years and above) Rural, urban, variety of neighborhood income groups
[32]	Ontario, Canada	Geriatric Care Physicians’ Perspectives on Providing Virtual Care: a Reflexive Thematic Synthesis of Their Online Survey Responses from Ontario, Canada	Mixed quantitative and qualitative online survey (n = 29)	Geriatric care physicians working in a long-term care home
[33]	Quebec, Canada	Implementing a Telehealth Support Tool for Community-Dwelling Older Adults During the COVID-19 Pandemic: A Qualitative Investigation of Provider Experiences	Qualitative: semi-structured online/phone interviews with health and social service providers (n = 20)	Healthcare providers who implemented the ESOGER telehealth tool for older adults
[34]	Ontario, Canada Quebec, Canada Alberta, Canada	Policy and Practices in Primary care that Supported the Provision and Receipt of Care for Older Persons During the COVID-19 Pandemic: a Qualitative Case Study in Three Canadian Provinces	Qualitative: case study. Interviews using telephone or video conferencing software (n = 64)	Primary care providers and older adult patients
[35]	Southwestern Ontario, Ontario Canada	Virtual care during COVID-19: The perspectives of older adults and their healthcare providers in a cardiac rehabilitation setting	Qualitative: semi-structured interviews (n = 15)	Older adults 65 years and older and cardiac rehabilitation HCPs from a cardiac rehabilitation unit
[36]	Eastern province of Canada	Older Adults’ Experiences with Remote Care for Specialized Health Service During the COVID-19 Pandemic: A Descriptive Qualitative Study	Qualitative: content analysis, semi-structured interviews (n = 21)	Patients 65 years of age and older, having received remote care (telephone or online video conference) from a specialist during the COVID 19 pandemic
[37]	London, Ontario, Canada	A Quantitative and Qualitative Study on Patient and Physician Perceptions of Nephrology Telephone Consultation During COVID-19	Qualitative & Quantitative: Email survey to patients and nephrologists (n = 235)	Adult patients (≥18 years) (77% ≥ 65 years old) with at least 1 nephrology telephone consultation during the pandemic Fully licensed nephrologists who transitioned to telephone consultation during the pandemic
[38]	Montreal, Quebec, Canada	Telemedicine in primary care of older adults: a qualitative study	Qualitative: semi structured interviews and focus groups (n = 29, n = 15)	Older adults aged 65 years and older and HCPs from McGill University family medicine sites and 4 Local Community Services Centres.
[35]	Canada	Personalized Telehealth: Redesigning Complex Care Delivery for the 65+ During the COVID Pandemic: a Survey of Patients, Caregivers, and Healthcare Providers	Qualitative and quantitative: electronic self administered or telephone-administered survey (n = 101)	Patients 65 years and older with multiple comorbidities, Healthcare providers, Caregivers from outpatient clinics
[39]	Greater Toronto Area, Ontario, Canada	“It’s better than nothing, but I do not find it to be ideal”: Older Adults’ Experience of TeleRehab During the First COVID-19 Lockdown	Qualitative: individual semi-structured interviews (n = 16)	Participants aged 60–85, with confirmed subjective cognitive problems
[40]	Toronto, Ontario, Canada	Barriers and Facilitators to Virtual Care in a Geriatric Medicine Clinic: A Semi-Structured Interview Study of Patient, Caregiver and Healthcare Provider Perspectives	Qualitative: semi-structured telephone interviews (n = 20)	Patients 65 years and older, healthcare providers, and caregivers from geriatric clinic
[41]	Canada	Socioeconomic Disparities in the Demand for and Use of Virtual Visits Among Senior Adults During the COVID-19 Pandemic: Cross-Sectional Study	Qualitative & Quantitative: cross-sectional web survey (n = 2303)	A subsample consisting of 2303 older adults, English and/or French speaking with internet access, from the Canadian Digital Health Survey

## Data Availability

Data sharing is not applicable. No new data were created or analyzed in this study.

## Supplementary Materials

The following supporting information can be downloaded at: https://www.mdpi.com/article/10.3390/healthcare13151937/s1, Table S1. Search terms; Table S2. Detailed search strings; Table S3. MMAT Results.

## Abbreviations

The following abbreviations are used in this manuscript:

PRISMA	Preferred Reporting Items for Systematic Reviews and Meta-Analysis
MMAT	Mixed Methods Appraisal Tool
OHIP	Ontario Health Insurance Plan

## References

[1] Koehn S., Habib S., Bukhari S. (2016). S4AC Case Study: Enhancing Underserved Seniors’ Access to Health Promotion Programs. Can. J. Aging Rev. Can. Vieil..

[2] Lai D.W.L., Surood S. (2010). Types and Factor Structure of Barriers to Utilization of Health Services among Aging South Asians in Calgary, Canada. Can. J. Aging Rev. Can. Vieil..

[3] Smith A.C., Thomas E., Snoswell C.L., Haydon H., Mehrotra A., Clemensen J., Caffery L.J. (2020). Telehealth for global emergencies: Implications for coronavirus disease 2019 (COVID-19). J. Telemed. Telecare.

[4] Government of Canada SC (2013). Statistics Canada: 2011 National Household Survey Profile. https://www12.statcan.gc.ca/nhs-enm/2011/dp-pd/prof/index.cfm?Lang=E.

[5] Bender J.L., Scruton S., Wong G., Abdelmutti N., Berlin A., Easley J., Liu Z.A., McGee S., Rodin D., Sussman J. (2024). Virtual follow-up care among breast and prostate cancer patients during and beyond the COVID-19 pandemic: Association with distress. Cancer Med..

[6] Zhao J., Zhang Z., Guo H., Li Y., Xue W., Ren L., Chen Y., Chen S., Zhang X. (2010). E-health in China: Challenges, Initial Directions, and Experience. Telemed. E-Health.

[7] Government of Canada SC (2018). Population Estimates on July 1, by Age and Gender. https://www150.statcan.gc.ca/t1/tbl1/en/tv.action?pid=1710000501.

[8] Glazier R.H., Green M.E., Wu F.C., Frymire E., Kopp A., Kiran T. (2021). Shifts in office and virtual primary care during the early COVID-19 pandemic in Ontario, Canada. Can. Med. Assoc. J..

[9] Stewart M., Ryan B. (2015). Ecology of health care in Canada. Can. Fam. Physician.

[10] Institute of Medicine (US) Committee on the Future Health Care Workforce for Older Americans (2008). Retooling for an Aging America: Building the Health Care Workforce.

[11] Chu C., Brual J., Fang J., Fleury C., Stamenova V., Bhattacharyya O., Tadrous M. (2022). The Use of Telemedicine in Older-Adults During the COVID-19 Pandemic: A Weekly Cross-Sectional Analysis in Ontario, Canada. Can. Geriatr. J..

[12] (2024). Get the Facts on Healthy Aging. https://www.ncoa.org/article/get-the-facts-on-healthy-aging/.

[13] Sierra-Heredia C., Tayyar E., Bozorgi Y., Thakore P., Hagos S., Carrillo R., Machado S., Peterson S., Goldenberg S., Wiedmeyer M.-L. (2024). Growing inequities by immigration group among older adults: Population-based analysis of access to primary care and return to in-person visits during the COVID-19 pandemic in British Columbia, Canada. BMC Prim. Care.

[14] Government of Canada SC (2021). Internet Use by Province and Age Group. https://www150.statcan.gc.ca/t1/tbl1/en/tv.action?pid=2210013501.

[15] Wang L., Guruge S., Montana G. (2019). Older Immigrants’ Access to Primary Health Care in Canada: A Scoping Review. Can. J. Aging Rev. Can. Vieil..

[16] Thomson M.S., Chaze F., George U., Guruge S. (2015). Improving Immigrant Populations’ Access to Mental Health Services in Canada: A Review of Barriers and Recommendations. J. Immigr. Minor. Health.

[17] Todd L., Harvey E., Hoffman-Goetz L. (2011). Predicting Breast and Colon Cancer Screening Among English-as-a-Second-Language Older Chinese Immigrant Women to Canada. J. Cancer Educ..

[18] Durbin A., Moineddin R., Lin E., Steele L.S., Glazier R.H. (2015). Mental health service use by recent immigrants from different world regions and by non-immigrants in Ontario, Canada: A cross-sectional study. BMC Health Serv. Res..

[19] Shaw J., Jamieson T., Agarwal P., Griffin B., Wong I., Bhatia R.S. (2017). Virtual care policy recommendations for patient-centred primary care: Findings of a consensus policy dialogue using a nominal group technique. J. Telemed. Telecare.

[20] Carman K.L., Dardess P., Maurer M., Sofaer S., Adams K., Bechtel C., Sweeney J. (2013). Patient And Family Engagement: A Framework For Understanding The Elements And Developing Interventions And Policies. Health Aff..

[21] Hardcastle L., Ogbogu U. (2020). Virtual care: Enhancing access or harming care?. Healthc. Manag. Forum.

[22] Garfan S., Alamoodi A., Zaidan B., Al-Zobbi M., Hamid R.A., Alwan J.K., Ahmaro I.Y., Khalid E.T., Jumaah F., Albahri O. (2021). Telehealth utilization during the COVID-19 pandemic: A systematic review. Comput. Biol. Med..

[23] Alsabeeha N.H., Atieh M.A., Balakrishnan M.S. (2023). Older Adults’ Satisfaction with Telemedicine During the COVID-19 Pandemic: A Systematic Review. Telemed. E-Health.

[24] Haimi M., Gesser-Edelsburg A. (2022). Application and implementation of telehealth services designed for the elderly population during the COVID-19 pandemic: A systematic review. Health Inform. J..

[25] Xie J.S., Nanji K., Khan M., Khalid M.F., Garg S.J., Thabane L., Sivaprasad S., Chaudhary V. (2022). Publication trends in telemedicine research originating from Canada. Healthc Manag. Forum.

[26] Elbaz S., Cinalioglu K., Sekhon K., Gruber J., Rigas C., Bodenstein K., Naghi K., Lavin P., Greenway K.T., Vahia I. (2021). A Systematic Review of Telemedicine for Older Adults With Dementia During COVID-19: An Alternative to In-person Health Services?. Front. Neurol..

[27] Page M.J., Moher D., Bossuyt P.M., Boutron I., Hoffmann T.C., Mulrow C.D., Shamseer L., Tetzlaff J.M., Akl E.A., Brennan S.E. (2021). PRISMA 2020 explanation and elaboration: Updated guidance and exemplars for reporting systematic reviews. BMJ (Clin. Res. Ed.).

[28] Sarker R., Roknuzzaman A.S.M., Hossain M.d.J., Bhuiyan M.A., Islam M.d.R. (2023). The WHO declares COVID-19 is no longer a public health emergency of international concern: Benefits, challenges, and necessary precautions to come back to normal life. Int. J. Surg..

[29] Hong Q.N., Fàbregues S., Bartlett G., Boardman F., Cargo M., Dagenais P., Gagnon M.-P., Griffiths F., Nicolau B., O’cAthain A. (2018). The Mixed Methods Appraisal Tool (MMAT) version 2018 for information professionals and researchers. Educ. Inf..

[30] Abdallah L., Stolee P., Lopez K.J., Whate A., Boger J., Tong C. (2022). The Impact of COVID-19 on Older Adults’ Perceptions of Virtual Care: Qualitative Study. JMIR Aging.

[31] Brual J., Chu C., Fang J., Fleury C., Stamenova V., Bhattacharyya O., Tadrous M. (2022). Virtual care use among older immigrant adults in Ontario, Canada during the COVID-19 pandemic: A repeated cross-sectional analysis. Geriatr. Med..

[32] Chuen V.L., Dholakia S., Kalra S., Watt J., Wong C., Ho J.M.-W. (2024). Geriatric care physicians’ perspectives on providing virtual care: A reflexive thematic synthesis of their online survey responses from Ontario, Canada. Age Ageing.

[33] Dassieu L., Develay E., Beauchet O., Quesnel-Vallée A., Godard-Sebillotte C., Tchouaket E., Puzhko S., Karunananthan S., Archambault P., Launay C. (2024). Implementing a Telehealth Support Tool for Community-Dwelling Older Adults During the COVID-19 Pandemic: A Qualitative Investigation of Provider Experiences. J. Aging Soc. Policy.

[34] Elliott J., Tong C., Gregg S., Mallinson S., Giguere A., Brierley M., Giosa J., MacNeil M., Juzwishin D., Sims-Gould J. (2023). Policy and practices in primary care that supported the provision and receipt of care for older persons during the COVID-19 pandemic: A qualitative case study in three Canadian provinces. BMC Prim. Care.

[35] Flores-Sandoval C., Sibbald S.L., Ryan B.L., Adams T.L., Suskin N., McKelvie R., Elliott J., Orange J.B. (2024). Virtual care during COVID-19: The perspectives of older adults and their healthcare providers in a cardiac rehabilitation setting. Can. J. Aging.

[36] Gaudine A., Parsons K., Smith-Young J. (2024). Older Adults’ Experiences with Remote Care for Specialized Health Service During the COVID-19 Pandemic: A Descriptive Qualitative Study. Can. J. Aging.

[37] Heyck Lee S., Ramondino S., Gallo K., Moist L.M. (2022). A Quantitative and Qualitative Study on Patient and Physician Perceptions of Nephrology Telephone Consultation During COVID-19. Can. J. Kidney Health Dis..

[38] Khanassov V., Ilali M., Ruiz A.S., Rojas-Rozo L., Sourial R. (2024). Telemedicine in primary care of older adults: A qualitative study. BMC Prim. Care.

[39] Rotenberg S., Oreper J.S., Bar Y., Davids-Brumer N., Dawson D.R. (2023). “It’s better than nothing, but I do not find it to be ideal”: Older adults’ experience of TeleRehab during the first COVID-19 lockdown. J. Appl. Gerontol..

[40] Watt J.A., Fahim C., Straus S.E., Goodarzi Z. (2022). Barriers and facilitators to virtual care in a geriatric medicine clinic: A semi-structured interview study of patient, caregiver and healthcare provider perspectives. Age Ageing.

[41] Yu E., Hagens S. (2022). Socioeconomic Disparities in the Demand for and Use of Virtual Visits Among Senior Adults During the COVID-19 Pandemic: Cross-sectional Study. JMIR Aging.

[42] Nene S., Rauch M., Belanger D., Bennett R., Berry G., Saad N., Wall M., Morais J.A., Morin S.N. (2023). Personalized Telehealth: Redesigning Complex Care Delivery for the 65+ During the COVID Pandemic: A Survey of Patients, Caregivers, and Health-care Providers. Can. Geriatr. J..

[43] Curran V.R., Hollett A., Peddle E. (2023). Patient Experiences With Virtual Care During the COVID-19 Pandemic: Phenomenological Focus Group Study. JMIR Form. Res..

[44] Gao H., Li R., Shen J., Yang H. (2025). Children’s gender and parents’ long-term care arrangements: Evidence from China. Appl. Econ..

[45] Perez F.P.P., Perez C.A., Chumbiauca M.N. (2022). Insights into the Social Determinants of Health in Older Adults. J. Biomed. Sci. Eng..

[46] Jeste D.V. (2022). Non-medical social determinants of health in older adults. Int. Psychogeriatr..

[47] Canada H. (2022). Enhancing Equitable Access to Virtual Care in Canada: Principle-Based Recommendations for Equity. https://www.canada.ca/en/health-canada/corporate/transparency/health-agreements/bilateral-agreement-pan-canadian-virtual-care-priorities-covid-19/enhancing-access-principle-based-recommendations-equity.html.

[48] Czaja S.J. (2017). The Potential Role of Technology in Supporting Older Adults. Public Policy Aging Rep..

[49] Gordon N.P., Hornbrook M.C. (2016). Differences in Access to and Preferences for Using Patient Portals and Other eHealth Technologies Based on Race, Ethnicity, and Age: A Database and Survey Study of Seniors in a Large Health Plan. J. Med. Int. Res..

[50] Government of Canada SC (2013). Household Access to the Internet at Home, by Household Income Quartile and Geography, Inactive. https://www150.statcan.gc.ca/t1/tbl1/en/tv.action?pid=2210000701.

[51] Cherid C., Baghdadli A., Wall M., Mayo N.E., Berry G., Harvey E.J., Albers A., Bergeron S.G., Morin S.N. (2020). Current level of technology use, health and eHealth literacy in older Canadians with a recent fracture—A survey in orthopedic clinics. Osteoporos Int..

[52] Gordon N.P., Hornbrook M.C. (2018). Older adults’ readiness to engage with eHealth patient education and self-care resources: A cross-sectional survey. BMC Health Serv. Res..

[53] Oh S.S., Kim K.A., Kim M., Oh J., Chu S.H., Choi J. (2021). Measurement of Digital Literacy Among Older Adults: Systematic Review. J. Med. Int. Res..

[54] Charles B.L. (2000). Telemedicine can lower costs and improve access. Healthc Financ. Manag..

[55] Gorenko J.A., Moran C., Flynn M., Dobson K., Konnert C. (2021). Social Isolation and Psychological Distress Among Older Adults Related to COVID-19: A Narrative Review of Remotely-Delivered Interventions and Recommendations. J. Appl. Gerontol..

[56] Aboujaoudé A., Bier N., Lussier M., Ménard C., Couture M., Demers L., Auger C., Pigot H., Caouette M., Lussier-Desrochers D. (2021). Canadian Occupational Therapists’ Use of Technology with Older Adults: A Nationwide Survey. OTJR Occup. Ther. J. Res..

[57] Czaja S.J., Charness N., Fisk A.D., Hertzog C., Nair S.N., Rogers W.A., Sharit J. (2006). Factors Predicting the Use of Technology: Findings From the Center for Research and Education on Aging and Technology Enhancement (CREATE). Psychol. Aging.

[58] Mannheim I., Schwartz E., Xi W., Buttigieg S.C., McDonnell-Naughton M., Wouters E.J.M., van Zaalen Y. (2019). Inclusion of Older Adults in the Research and Design of Digital Technology. Int. J. Environ. Res. Public Health.

[59] Bhome R., Huntley J., Dalton-Locke C., Juan N.V.S., Oram S., Foye U., Livingston G. (2021). Impact of the COVID-19 pandemic on older adults mental health services: A mixed methods study. Int. J. Geriatr. Psychiatry.

[60] Canada Health Infoway (2024). Infoway Insights: National Survey of Canadian Physicians. https://insights.infoway-inforoute.ca/national-physician-survey.

[61] Nägle S., Schmidt L. (2012). Computer acceptance of older adults. Work J. Prev. Assess Rehabil..

[62] Appleton R., Williams J., Juan N.V.S., Needle J.J., Schlief M., Jordan H., Rains L.S., Goulding L., Badhan M., Roxburgh E. (2021). Implementation, Adoption, and Perceptions of Telemental Health During the COVID-19 Pandemic: Systematic Review. J. Med. Int. Res..

[63] Lieneck C., Weaver E., Maryon T. (2021). Outpatient Telehealth Implementation in the United States during the COVID-19 Global Pandemic: A Systematic Review. Medicina.

[64] Canada H. (2022). Virtual Care Policy Framework. https://www.canada.ca/en/health-canada/corporate/transparency/health-agreements/bilateral-agreement-pan-canadian-virtual-care-priorities-covid-19/policy-framework.html.

