# Supplementary material for: Virtual Care Perceptions and Experiences of Older Adults During COVID-19 in Canada: A Systematic Review
Source: Healthcare (Basel). 2025 Aug 7;13(15):1937. doi: 10.3390/healthcare13151937 (PMC12345755; doi:10.3390/healthcare13151937)
Supplement: Supplementary file 1 [file healthcare-13-01937-s001.zip › Supplementary File S1. Detailed Search Strategy.pdf]

Detailed Search Strategy

Table S1. Search terms

Virtual Care, Virtual Medicine, Telehealth, Telemedicine, Telecare, Mobile Health, mHealth, eHealth, Telerehabilitation
COVID-19, SARS-CoV-2, coronavirus, COVID, Covid 19 Pandemic
Barrier, Disparity, Challenge
Canada
Older adult, 65 years and older

Table S2. Detailed search strings

**Database**	**Search String**	**Results** *Search date: May 25, 2025*
Pubmed	("Telemedicine"[MeSH] OR "Remote Consultation"[MeSH] OR "Telehealth" OR "Virtual Care" OR "Virtual Medicine" OR "Telecare" OR "Mobile Health" OR "mHealth" OR "eHealth" OR "Digital Health" OR "Telerehabilitation")<br>AND<br>("COVID-19"[MeSH] OR "SARS-CoV-2"[MeSH] OR Coronavirus OR "COVID" OR "Covid 19 Pandemic")<br>AND<br>("Health Services Accessibility"[MeSH] OR "Health Equity"[MeSH] OR Barrier* OR Disparit* OR Challenge* OR Inequalit* OR Access)<br>AND<br>("Canada"[MeSH] OR Canada OR Ontario OR Quebec OR Alberta OR "British Columbia" OR "Nova Scotia" OR "New Brunswick" OR "Saskatchewan" OR Manitoba OR "Prince Edward Island" OR Newfoundland OR Labrador)<br>AND<br>("Aged"[MeSH] OR "Older Adults" OR "Older adult*" OR Elderly OR Seniors OR Geriatric* OR "65 years and older")	232

Scopus	(TITLE-ABS-KEY (("Telehealth" OR "Virtual Care" OR "Virtual Medicine" OR "Telemedicine" OR "Telecare" OR "Mobile Health" OR "mHealth" OR "eHealth" OR "Digital Health" OR "Telerehabilitation")) AND TITLE-ABS-KEY (("COVID-19" OR "Coronavirus" OR "SARS-CoV-2" OR "COVID")) AND TITLE-ABS-KEY (("Barrier*" OR "Disparit*" OR "Challenge*" OR "Inequalit*" OR "Access" OR "Health Equity" OR "Health Services Accessibility")) AND TITLE-ABS-KEY (("Canada" OR "Ontario" OR "Quebec" OR "British Columbia" OR "Nova Scotia")) AND TITLE-ABS-KEY (("Older adult*" OR "Elderly" OR "Seniors" OR "Geriatric*" OR "65 years and older")))	52
EBSCOhost	((MH "Telemedicine+") OR "telemedicine" OR "telehealth" OR "virtual care" OR "virtual medicine" OR "telecare" OR "eHealth" OR "mHealth" OR "mobile health" OR "digital health" OR "telerehabilitation")<br>AND<br>((MH "Coronavirus Infections+") OR "COVID-19" OR "COVID" OR "SARS-CoV-2" OR "Coronavirus" OR "Covid 19 pandemic")<br>AND<br>((MH "Health Services Accessibility+") OR (MH "Health Equity") OR barrier* OR disparit* OR challenge* OR inequalit* OR access)<br>AND<br>("Canada" OR "Ontario" OR "Quebec" OR "British Columbia" OR "Alberta" OR "Nova Scotia" OR "Newfoundland" OR "New Brunswick" OR "Saskatchewan" OR "Manitoba" OR "Prince Edward Island" OR "Labrador")<br>AND<br>((MH "Aged+") OR "older adult*" OR elderly OR seniors OR geriatric* OR "65 years and older")	71
Web of Science	TS=("telemedicine" OR "telehealth" OR "virtual care" OR "virtual medicine" OR "telecare" OR "eHealth" OR "mHealth" OR "mobile health" OR "digital health" OR "telerehabilitation")<br>AND<br>TS=("COVID-19" OR "COVID" OR "SARS-CoV-2" OR "Coronavirus" OR "Covid 19 pandemic")<br>AND<br>TS=("barrier*" OR "disparit*" OR "challenge*" OR "inequalit*" OR "access" OR "health equity" OR "health services accessibility")<br>AND<br>TS=("Canada" OR "Ontario" OR "Quebec" OR "British	44

	Columbia" OR "Alberta" OR "Nova Scotia" OR "Newfoundland" OR "New Brunswick" OR "Saskatchewan" OR "Manitoba" OR "Prince Edward Island" OR "Labrador")<br>AND<br>TS=("older adult*" OR "elderly" OR "seniors" OR "geriatric*" OR "65 years and older")<br><br>Excluding pre-prints	
