# Supplementary material for: Virtual Care Perceptions and Experiences of Older Adults During COVID-19 in Canada: A Systematic Review
Source: Healthcare (Basel). 2025 Aug 7;13(15):1937. doi: 10.3390/healthcare13151937 (PMC12345755; doi:10.3390/healthcare13151937)
Supplement: Supplementary file 1 [file healthcare-13-01937-s001.zip › Supplementary File S2. MMAT Results.pdf]

MMAT Results

Table S3. MMAT Results

	**Screening Questions**		**Qualitative**					**Quantitative/ Descriptive**				
**Study**	Are there clear research questions?	Do the collected data allow to address the research questions?	1.1. Is the qualitative approach appropriate to answer the research question?	1.2. Are the qualitative data collection methods adequate to address the research question?	1.3. Are the findings adequately derived from the data?	1.4. Is the interpretation of results sufficiently substantiated by data?	1.5. Is there coherence between qualitative data sources, collection, analysis and interpretation?	4.1. Is the sampling strategy relevant to address the research question?	4.2. Is the sample representative of the target population?	4.3. Are the measurements appropriate?	4.4. Is the risk of nonresponse bias low?	4.5. Is the statistical analysis appropriate to answer the research question?
Abdullah et al. 2022	Yes	Yes	Yes	Yes	Yes	Yes	Yes	N/A	N/A	N/A	N/A	N/A
Bender et al. 2024	Yes	Yes	N/A	N/A	N/A	N/A	N/A	Yes	Somewhat	Yes	Unclear	Yes
Brual J. et al, 2023	Yes	Yes	N/A	N/A	N/A	N/A	N/A	Yes	No	Yes	Yes	Yes
Chu C et al. 2022	Yes	Yes	N/A	N/A	N/A	N/A	N/A	Yes	Yes	Yes	Yes	Yes
Chuen V. et al, 2024	Yes	Yes	Yes	Yes	Yes	Yes	Yes	N/A	N/A	N/A	N/A	N/A
Dassieu L et al. 2022	Yes	Yes	Yes	Yes	Yes	Yes	Yes	N/A	N/A	N/A	N/A	N/A

Elliot J. et al, 2023	Yes	Yes	Yes	Yes	Yes	Yes	Yes	N/A	N/A	N/A	N/A	N/A
Flores-Sandoval1 et al., 2024	Yes	Yes	Yes	Yes	Yes	Yes	Yes	N/A	N/A	N/A	N/A	N/A
Gaudine A. et al, 2023	Yes	Yes	Yes	Yes	Yes	Yes	Yes	N/A	N/A	N/A	N/A	N/A
Heyck Lee S. et al, 2022	Yes	Yes	N/A	N/A	N/A	N/A	N/A	Yes	Yes	Yes	No	Yes
Khanassov et al., 2024	Yes	Yes	Yes	Yes	Yes	Yes	Yes	N/A	N/A	N/A	N/A	N/A
Nene S. et al, 2023	Yes	Yes	Yes	Yes	Yes	Yes	Yes	N/A	N/A	N/A	N/A	N/A
Rotenberg S. et al, 2023	Yes	Yes	Yes	Yes	Yes	Yes	Yes	N/A	N/A	N/A	N/A	N/A
Watt J. et al, 2022	Yes	Yes	Yes	Yes	Yes	Yes	Yes	N/A	N/A	N/A	N/A	N/A
Yu E. et al, 2022	Yes	Yes	N/A	N/A	N/A	N/A	N/A	Yes	No	Yes	Unclear	Yes
